# Genetic determinants of tendon and ligament injuries in athletes: a narrative review from single variants to gene-gene interactions

**DOI:** 10.3389/fsysb.2026.1858521

**Published:** 2026-09-23

**Authors:** Amina Marrouh, Arij El Haddouchi, Souad Kartti, Elmostafa El Fahime, Saber Boutayeb, Younes Chagar, Christophe Baudot, Lahcen Belyamani, Taoufiq Dakka, Rachid Eljaoudi

**Affiliations:** 1 Laboratory of Drug Sciences Research, Mohammed VI Faculty of Pharmacy, Mohammed VI University of Sciences and Health (UM6SS), Casablanca, Morocco; 2 Mohammed VI University of Sciences and Health (UM6SS), Casablanca, Morocco; 3 Mohammed VI Center for Research and Innovation (CM6RI), Rabat, Morocco; 4 Interdisciplinary Laboratory of Biotechnology and Health, Mohammed VI Higher School of Engineers in Health Sciences, Mohammed VI University of Sciences and Health (UM6SS), Casablanca, Morocco; 5 Sports and Health Unit, Mohammed VI Foundation of Science and Health (FM6SS), Rabat, Morocco; 6 Rabat Medical and Pharmacy School, Mohammed V University, Rabat, Morocco

**Keywords:** anterior cruciate ligament (ACL), gene-gene interaction, genetic polymorphism, GWAS, sports injury, tendinopathy

## Abstract

Tendon and ligament injuries are common in athletes and can result in significant functional impairment. Genetic factors are increasingly recognized as key contributors to individual susceptibility. A structured narrative literature search was conducted in PubMed, Scopus, Web of Science, and Google Scholar to identify studies investigating genetic polymorphisms associated with tendon and ligament injuries in athletic populations. Eligible studies published in English and involving elite, professional, recreational, or physically active individuals were narratively synthesized. This review synthesizes current evidence on polymorphisms associated with these injuries, identifying 70 genes and 107 variants across candidate-gene studies, haplotype analyses, and genome-wide association studies (GWAS). Specifically, the evidence included 33 candidate genes with 43 polymorphisms, 25 polymorphisms evaluated in haplotype analyses involving these candidate genes as well as three additional genes, and 34 genes identified through GWAS encompassing 39 variants. These genes are involved in extracellular matrix (ECM) organization, inflammation, tissue remodeling, angiogenesis, and muscle function. Structural genes, particularly collagens (*COL1A1, COL3A1, COL5A1*), proteoglycans (*ACAN, DCN, BGN*), matrix remodeling (*MMPs, TIMP2*), and glycoproteins (*FMOD, TNC*), as well as genes related to inflammatory (*IL1B, IL6, TNF-*α), and vascular pathways (*VEGFA, VEGFR2)*, were frequently implicated. Importantly, gene-gene interactions and haplotypes appear to be more informative than single polymorphisms for explaining injury risk. Although GWAS studies remain limited, they have identified novel loci, reinforcing the polygenic nature of these conditions. Overall, these findings highlight the complexity of genetic influences and support integrating genetic, biomechanical, and environmental factors to improve personalized prevention and rehabilitation strategies in sports medicine.

## Highlights

- This review provides a comprehensive synthesis of 70 genes and 107 variants associated with tendon and ligament injuries in athletes, including 33 candidate genes with 43 polymorphisms, 25 polymorphisms evaluated in haplotype analyses involving these candidate genes as well as three additional genes, and 34 genes identified through GWAS encompassing 39 variants, thereby integrating evidence across multiple biological pathways.
- It highlights the importance of gene-gene interactions and haplotype structures, which may better explain injury susceptibility than single genetic variants alone.
- It consolidates emerging GWAS findings and emphasizes the polygenic nature of these injuries, supporting more integrative approaches to injury risk assessment and prevention in sports medicine.

## 1 Introduction

Tendon and ligament injuries account for over 30% of sports-related injuries, impacting both amateur and professional athletes (Sancerne and Kaux, 2015). Among these injuries, tendon ruptures, particularly Achilles tendon ruptures, are the most common. In the United States, 60.1% of tendon ruptures are sports-related, with an average annual increase of 1.9% (Lyons et al., 2024). In football, the incidence of these injuries was markedly higher during matches than during training, with rates approximately 7.8-fold higher in professional footballers and 5.4-fold higher in amateur players (Gurau et al., 2023).

Understanding the genetic factors that influence susceptibility to these injuries is important for improving our knowledge of individual variability in injury risk and for supporting the development of more personalized training and prevention strategies. Specific gene polymorphisms have been linked to athletic performance and vulnerability to these injuries (El Haddouchi et al., 2025; Maffulli et al., 2013; Marrouh et al., 2025; Varillas-Delgado, 2024). Certain genetic variations affect physical performance, recovery, and the composition of the extracellular matrix (ECM), which in turn impacts the integrity of tendons and ligaments (İpekoğlu et al., 2023; M et al., 2020; Jacob et al., 2022; Briški et al., 2021; Leźnicka et al., 2021).

Although previous reviews have addressed this topic (Maffulli et al., 2013; September et al., 2007), numerous studies have since identified new polymorphisms and refined our understanding of the underlying genetic mechanisms (Varillas-Delgado, 2024; Jacob et al., 2022; Ebert et al., 2023). However, important gaps remain in the existing literature. In particular, most previous reviews did not fully incorporate recent genome-wide association studies (GWAS), haplotype-based analyses, or more comprehensive multi-locus approaches. Moreover, limited attention has been given to the distinction between tendon and ligament injuries, despite their distinct biological and biomechanical characteristics. In addition, existing evidence has not been systematically stratified by key determinants, such as sport type, sex, competitive level, or specific injury subtype, which may influence genetic susceptibility. This lack of detailed stratification limits the clinical and translational applicability of previous syntheses.

Furthermore, it is important to emphasize that different injury phenotypes, including tendinopathy, tendon rupture, ligament injury, anterior cruciate ligament (ACL) rupture, hamstring injury, and ankle injury, represent distinct clinical and biological entities. These conditions may involve different pathophysiological processes and potentially distinct genetic determinants. Therefore, genetic associations should be interpreted within the specific context of the investigated injury phenotype, rather than generalized across all musculoskeletal injuries (September et al., 2007; Hall et al., 2022; Larruskain, 2018; Pruna et al., 2013). A conceptual framework summarizing the biological pathways through which genetic variants may influence tendon and ligament injury susceptibility in athletes (Figure 1).

**FIGURE 1:**
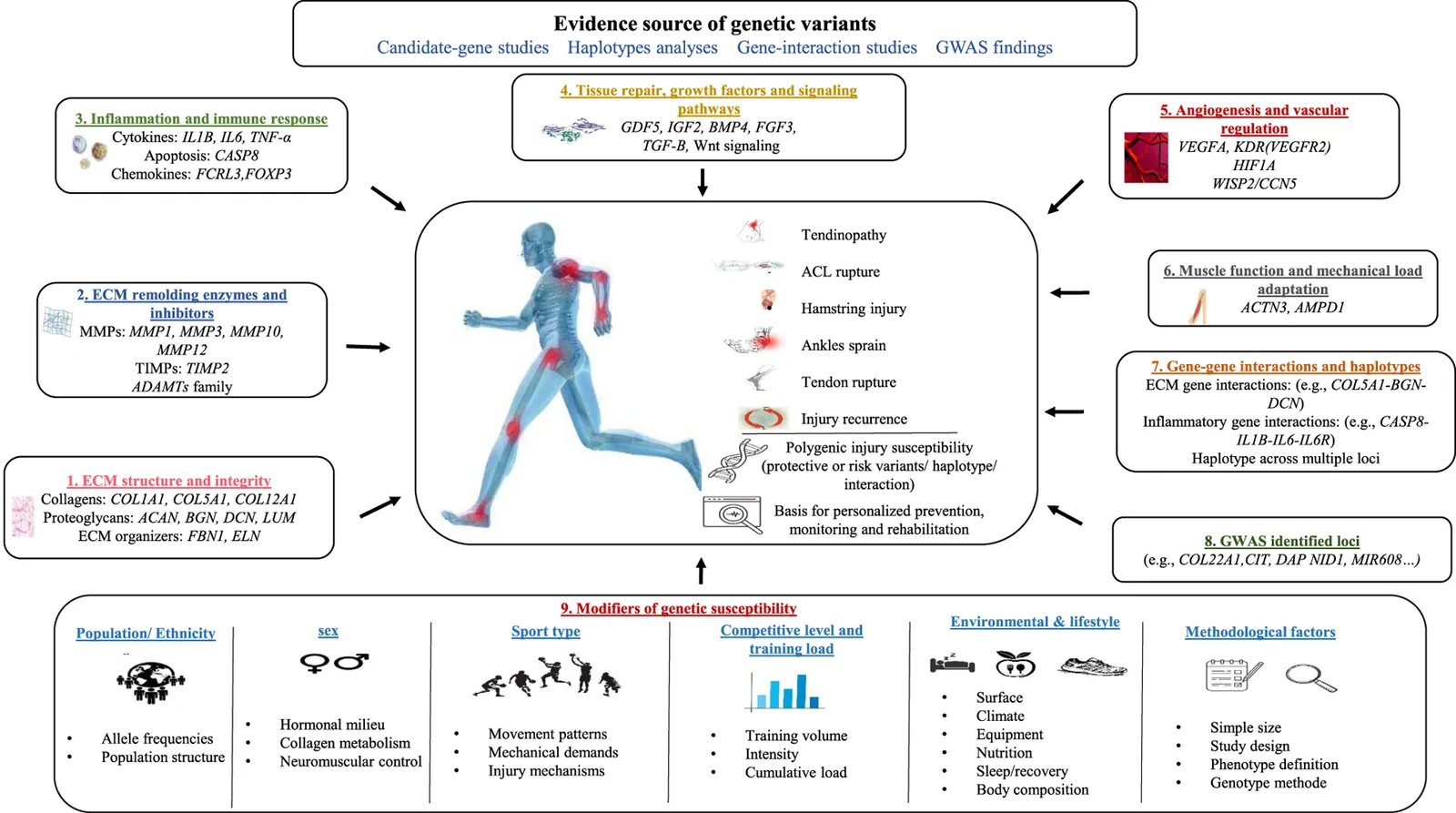
Conceptual framework summarizing the biological pathways through which genetic variants may influence tendon and ligament injury susceptibility in athletes. Genes identified through candidate-gene studies, haplotype analyses, gene-gene interaction studies, and GWAS were grouped according to their principal biological functions. These pathways converge to influence tissue homeostasis, repair capacity, and adaptation to mechanical loading, ultimately contributing to injury susceptibility. The magnitude of genetic effects may be modified by sex, ethnicity, sport type, competition level, training load, environmental factors, and methodological differences among studies.

This review stands out by integrating the most recent data and focusing exclusively on studies conducted in athletes, enabling a more targeted and relevant analysis within the context of sports medicine research. By consolidating these findings, the review serves as a valuable reference for guiding future research and the development of personalized prevention strategies. In this context, this narrative review is structured around three complementary objectives. First, it aims to systematically identify and describe genes and polymorphisms associated with tendon and ligament injuries in athletes. Second, it seeks to synthesize these genetic variants by organizing them according to their roles in key biological pathways, including ECM remodeling, collagen structure, and musculoskeletal adaptation. Third, it provides an interpretive analysis by critically evaluating the consistency and robustness of the available evidence across studies and discussing its relevance to injury susceptibility and personalized prevention strategies in sports medicine. By integrating the most recent data and focusing exclusively on studies conducted in athletes, this review offers a more targeted and clinically relevant synthesis of current knowledge. It aims to consolidate existing findings, serve as a reference for future research, and identify new avenues for investigation in sports genetics.

## 2 Review methodology

This narrative review used a structured literature search to identify studies examining genetic factors associated with tendon and ligament injuries in athletes and physically active populations. Electronic searches were conducted in PubMed, Scopus, Web of Science, and Google Scholar to retrieve studies published through April 2026. The search strategy combined keywords and Medical Subject Headings (MeSH) related to sports injuries and genetics, including “tendon injury”, “ligament injury”, “anterior cruciate ligament”, “ACL rupture”, “tendinopathy”, “sports injury”, “athletes”, “genetic polymorphism”, “single nucleotide polymorphism”, “SNP”, “haplotype”, “gene-gene interaction”, and “genome-wide association study” or “GWAS”.

Studies were considered eligible if they: (1) investigated associations between genetic variants and tendon or ligament injuries; (2) included elite, professional, semi-professional, recreational athletes, or physically active individuals; and (3) were published in English. Both candidate-gene association studies and genome-wide association studies were included. Reviews, editorials, conference abstracts lacking sufficient data, animal studies, and studies unrelated to musculoskeletal sports injuries were excluded. Studies involving mixed populations were retained only when data related to athletes or physically active participants were clearly identifiable and relevant to the objectives of the review.

For this review, the term “athletes” was broadly defined to encompass individuals engaged in organized sports or regular physical training at different competitive levels and across various sports disciplines. Consequently, the included studies represented a heterogeneous range of populations, including football players, volleyball players, runners, skiers, and other sport-specific cohorts, as well as control groups with varying levels of physical activity. Because genetic associations may vary by sport discipline, competitive level, sex, ethnicity, training exposure, and injury mechanism, this heterogeneity was considered in data interpretation and acknowledged as a potential source of variability across studies.

The screening and selection process was based on the relevance of titles, abstracts, and full texts to the review’s objectives. Extracted data included the investigated genes and polymorphisms, study population characteristics, sport discipline, injury type, reported associations, and the biological pathways potentially involved in injury susceptibility. The identified genetic factors were subsequently classified according to their principal biological functions, including ECM organization, inflammation, tissue remodeling, angiogenesis, oxygen regulation, and muscle function.

## 3 Genetic polymorphisms related to tendon and ligament injuries in athletes

Numerous genetic variants have been identified in relation to tendon and ligament injuries in athletes, with 30 genes and 43 polymorphisms associated with these injuries, as summarized in Table 1. Collectively, these findings underscore the relevance of gene-gene interactions and haplotype structures, rather than single polymorphisms alone, in modulating injury susceptibility (3 genes and 7 polymorphisms) (Table 2). These genetic variants influence the function of expressed proteins, particularly in the repair of these tissues following injury. This includes mutations that affect the expression levels of chemokines and cytokines, which are crucial for initiating the inflammatory response and recruiting immune cells to the injury site (Rahim et al., 2017; Lulińska-Kuklik et al., 2019a). Moreover, disruption of proteins involved in maintaining the structure, integrity, and elasticity of tendons and ligaments can also contribute to increased susceptibility to injury (Pruna et al., 2013). Additionally, genes encoding proteoglycans, glycoproteins, and other ECM components may further enhance the vulnerability of tendons and ligaments to injury (Cięszczyk et al., 2017).

**TABLE 1:** Genetic polymorphisms associated with tendon and ligament injuries in athletes.

Genes	Full name	Polymorphisms	Localization	Injury phenotype	Alleles/Genotypes associated with increased injury risk	Alleles/Genotypes associated with decreased injury risk	Evidence level
*ACAN*	Aggrecan	rs1516797	15q26.1	ACL rupture	NA	TT (Cięszczyk et al., 2017)	Moderate
*ACTN3*	Actinin alpha 3	rs1815739	11q13.2	Achilles tendon injuries	RR (Gutiérrez-Hellín et al., 2021)	XX (Kim et al., 2014)	Weak
*ADAMTS14*	ADAM metallopeptidase with thrombospondin type 1 motif 14	rs4747096	10q22.1	Hamstring injuries	AG (Larruskain, 2018)	NA	Weak
*AMPD1*	Adenosine monophosphate deaminase 1	rs17602729	1p13.2	Ankle and knee injuries	T (Varillas-Delgado, 2024; McCabe and Collins, 2018)	CC (Varillas-Delgado, 2024; McCabe and Collins, 2018)	Moderate
*BGN*	Biglycan	rs1042103	Xq28	ACL rupture	A (Cięszczyk et al., 2017)	NA	Moderate
*BMP4*	Bone morphogenetic protein 4	rs2761884	14q22.2	Tendinopathy	*T* (Salles et al., 2015)	*NA*	Weak
*CASP8*	Caspase 8	rs3834129 rs1045485	2q33-34	Ligament injuries TEN	Ins (Rahim et al., 2017) GC (Rahim et al., 2017)	NA NA	Moderate
*COL12A1*	Collagen type XII alpha 1 chain	rs240736 rs970547	6q13-q14.1	ACL rupture	AA (Posthumus et al., 2010) AA (O’Connell et al., 2015)	NA NA	Strong
*COL1A2*	Collagen type I alpha 2 chain	rs42524 rs2621215	7q21.3	ACL rupture KLR	C (Krawczak-Wójcik et al., 2025) G (Krawczak-Wójcik et al., 2025)	NA	Moderate
*COL3A1*	Collagen type III alpha 1 chain	rs1800255	2q32.2	ACL rupture	AA (Stępień-Słodkowska et al., 2015a; O’Connell et al., 2015)	NA	Moderate
*COL5A1*	Collagen type V alpha 1 chain	rs12722	9q34.3	ACL rupture Hamstring injuries Ankle injuries	T (McCabe and Collins, 2018)	CC (Calò et al., 2024)	Strong
*COLGAT1/GLT25D1*	Collagen Beta(1-O)Galactosyltransferase 1	rs8090	19p13.11	ACL rupture	NA	AA (Brazier et al., 2023)	Weak
*DCN*	Decorin	rs516115	12q21.33	Hamstring injuries, ACL rupture	A (Larruskain, 2018)	GG (Mannion et al., 2014)	Moderate
*EMILIN1*	EMILIN1	rs2289360 (G/T, A/T, C/T)	2p23.3	Ligament injuries TEN	A (Pruna et al., 2013; Artells et al., 2016; Lulińska et al., 2023; Pruna et al., 2015) G (Jacob et al., 2022) C (Hall et al., 2022)	T (Hall et al., 2022)	Weak
*FCRL3*	Fc receptor like 3	rs7528684 (−169T>C)	1q23.1	Tendinopathy	C (Salles et al., 2018)	NA	Weak
*FBN2*	Fibrillin 2	rs331079	5q23.3	Tendon injury	GG (Khoury et al., 2015)	NA	Weak
*FMOD*	Fibromodulin	rs7543148	1q32.1	ACL rupture	NA	TT (Lulińska et al., 2023)	Moderate
*FOXP3*	Forkhead box P3	rs3761549 (−2383C>T)	Xp11.23	Tendinopathy	T (Salles et al., 2018)	NA	Weak
*GDF5*	Growth differentiation factor 5	rs143383	20q11.22	Ankle and knee injuries	TT (McCabe and Collins, 2018)	NA	Weak
*HIF1A*	Hypoxia inducible factor 1 subunit alpha	rs11549465	14q23.2	Hamstring injuries	CC (Larruskain, 2018)	NA	Weak
*IGF2*	Insulin-like growth factor 2	rs680 rs3213221	GG (McCabe and Collins, 2018) CC (Jacob et al., 2022)	Ligament injuries TEN	NA GC (Pruna et al., 2013)	NA	Moderate
*IL1B*	Interleukin-1βeta	rs16944	2q14.1	ACL rupture	TT (Rahim et al., 2017)	CT (Lorenz et al., 2024)	Moderate
*IL6*	Interleukin- 6	rs1800795	7p15.3	ACL rupture	GG (Larruskain, 2018; Lulińska-Kuklik et al., 2019a)	CC (Lorenz et al., 2024)	Moderate
*KDR/VEGFR2*	Vascular endothelial growth factor receptor 2	rs2071559 (−604C>T) rs2239702 (1192G>A)	4q12	Tendinopathy, ACL rupture	GG (Salles et al., 2016; Rahim et al., 2014)	AG (Salles et al., 2016; Rahim et al., 2014) A (Salles et al., 2016)	Weak
*MMP1*	Matrix metallopeptidase 1	rs1799750	11q22.2	ACL rupture	II (Łosińska et al., 2025)	ID (Łosińska et al., 2025)	Weak
*MMP10*	Matrix metallopeptidase 10	rs486055	11q22.2	ACL rupture	T and CT (Łosińska et al., 2025)	NA	Weak
*MMP12*	Matrix metallopeptidase 12	rs2276109	11q22.2	ACL rupture	G (Łosińska et al., 2025)	NA	Weak
*TIMP2*	TIMP metallopeptidase inhibitor 2	rs4789932	17q25.3	Achilles tendon injuries	TT (El Khoury et al., 2013)	NA	Weak
*TNC*	Tenascin C	rs2104772	9q33.1	Achilles tendon injuries TEN Hamstring injury	A (Larruskain, 2018)	NA	Moderate
*TNF-*α	Tumor necrosis factor	rs1800629 (−308 G/A)	6p21.33	Tendinopathies	A (Lopes et al., 2021)	NA	Moderate
*VEGFA*	Vascular endothelial growth factor A	rs35569394 rs1570360 rs2010963	6p21.1	ACL rupture	I (M et al., 2020) NA C (Lulińska-Kuklik et al., 2019b)	NA GA (Rahim et al., 2014) NA	Strong

The polymorphisms rs699947 (-2578C>A), rs679620, rs650108, rs591058, and rs1800012 (+1245C/A) are not presented in the table due to conflicting results for these variants. Please refer to the paragraphs below for more details. Evidence levels (strong, moderate, weak) were assigned based on study replication, consistency of findings across populations, sample size, and availability of meta-analytic support. Strong evidence indicates replicated and consistent associations, moderate evidence reflects limited or partially inconsistent replication, and weak evidence corresponds to preliminary or non-replicated findings. ACL: anterior cruciate ligament, KLR: knee ligament rupture, TEN: tendon injuries, NA: not available.

**TABLE 2:** Gene-gene interactions and haplotype combinations associated with susceptibility to tendon and ligament injuries in athletes.

Study	Study design	Population	Sport	Sex	Sample size	Injury type	Competitive level	Gene-genes interaction	Polymorphisms interaction	Haplotypes/pseudo-haplotypes	Main genetic effect
Mannion et al. (2014)	Case-control	South Africa	Contact sports, Non-contact jumping sports, Non-contact non-jumping sports, and Skiing sports	Males	227 case 234 CON	ACL ruptures	Not reported	*ACAN*	(rs2351491- rs1042631- rs1516797)	T-C-T	Increased risk
								*BGN*	(rs1042103-rs1126499)	G-C	Protection effect
								*LUM-DCN*	(rs2268578 -rs13312816- rs516115)	T-A-G	Protective effect
Cięszczyk et al. (2017)	Case-control	Poland	Contact sports, Non-contact jumping sports, Non-contact non-jumping sports, and Skiing sports	Males and females	229 case 143 CON	ACL ruptures	Polish 1st division soccer players (10–12 h/week)	*BGN*	(rs1042103-rs1126499)	C-A T-A	Increased risk Increased risk
Willard et al. (2018)	Case- control	South Africa	Not specified	Males	227 case 234 CON	ACL ruptures	Not reported	*BGN-COL5A1-DCN*	(rs1126499-rs1042103- rs12722- rs516115)	C-G-C-G C-A-T-A T-A-T-A T-G-T-A	Protective effect Protective effect Increased risk Increased risk
Salles et al. (2015)	Case-control	Brazil	Volleyball	Males	52 case 86 CON	Tendinopathy	Competitive volleyball athletes (≈13 years’ experience)	*BMP4*	(rs2761884-rs17563-rs2855529-rs2071047-rs762642)	T-T-G-G-A	Increased risk
Rahim et al. (2017)	Case-control	South Africa	Contact sports, Non-contact jumping sports, Non-contact non-jumping sports, and Skiing sports	Males	171 case 232 CON	ACL	Mixed sports participants	*CASP8*	(rs3834129 -rs1045485)	Del-G	Increased risk
Seale et al. (2020)	Case-control	South Africa	Contact sports, Non-contact jumping sports, Non-contact non-jumping sports, and Skiing sports	Males	171 case 232 CON	ACL	Mixed sports participants	*CASP8* *COL5A1-IL1B-IL6-IL6R*	(rs3834129-rs1045485-rs13113)	Ins-G-T Ins-G-A	Increased risk Protective effect
									(rs3834129-rs13113) (rs1045485-rs13113)	Ins-T	Increased risk
									(rs3834129-	G-T	Increased risk
									rs1045485) (rs12722- rs16944- rs1800795- rs2228145)	Del-C T-C-G-A	Protective effect Protective effect
Ficek et al. (2013)	Case-control	Poland	Soccer	Males	91 case 143 CON	ACL	Polish 1st division soccer players	*COL1A1*	(rs1107946- rs1800012)	G-T	Protective effect
Seale et al. (2020)	Case-control	South Africa, Sweden	Swimming, triathlon, water polo	Males and females	3 cohorts (100 RCT 100 CON) (102 ACL 111CON) (102 CTS 148 CON)	RCT, ACL, CTS	Competitive athletes	*CASP8*	(rs3834129-rs1045485-rs13113)	Ins-G-T Ins-G-A	Increased risk Protective effect
									(rs3834129-rs13113) (rs1045485-rs13113)	Ins-T	Increased risk
									(rs3834129-rs1045485)	G-T	Increased risk
									(rs7932320-rs1893047- rs12574452-	Del-C	Protective effect Increased risk
								*FGF3*	rs4631909- rs4980700)	T-G-G-T-A	
Perini et al. (2022)	Case-control	Not specified	Multiple sports	Male and females	146 Case 192 CON	ACL	High training load athletes	*COL1A1-COL1A2*	(rs1107946-rs412777- rs42524-rs2621215)	GT-AA-GC-TT TT-AA-GC-TT	Protective effect Protective effect
Lulińska-Kuklik et al. (2018)	Case-control	Poland	Soccer	Males	134 ACL 211 CON	ACL	Polish professional soccer players. All participants were soccer players	*COL5A1*	(rs12722-rs13946)	C-C	Protective effect
O’Connell et al. (2015)	Case-control	South Africa, Poland	Multiple sports	Males and females	333 ACL 378 CON	ACL	Competitive athletes	*COL5A1-COL12A1*	(rs12722- rs970547)	T-A	Increased risk
Salles et al. (2016)	Case- control	Brazil	Volleyball	Males and females	88 Case 91 CON	Tendinopathy	Competitive volleyball players	*KDR*	(-604C>T, 1192G>A, 1719T>A)	C-G-A C-A-T	Protective effect Protective effect
Rahim et al. (2014)	Case-control	South Africa	Contact sports, Non-contact jumping sports, Non-contact non-jumping sports, and Skiing sports	Males and females	227 ACL 227 CON	ACL	Mixed sports athletes	*KDR*	(rs1870377, rs2071559)	G-A	Increased risk
								*VEGFA*	(rs699947- rs1570360-rs2010963)	A-A-G C-G-C	Protective effect Increased risk
Posthumus et al. (2012)	Case- control	South Africa	Contact sports, non-contact jumping sports, noncontact non-jumping sports and skiing sport	Males and females	129 ACL 216 CON	ACL	Mixed sports athletes	*MMP10-MMP1-MMP3-MMP12*	(rs486055- rs1799750- rs679620- rs2276109)	C-2G-G-G	Protective effect
Raleigh et al. (2009)	Case-control	Caucasian	Multiple sports	Males	114 Case 98 CON	Achilles tendinopathy	Mixed sports athletes	*MMP3*	(rs679620, rs591058, rs650108)	A-T-G	Increased risk
Briški et al. (2021)	Case-control	Caucasian	Soccer, handball, basketball, martial arts, skiing	Males and females	63 Case 92 CON	Achilles tendinopathy	Mixed sports athletes	*MMP3*	(rs679620, rs591058, rs650108)	G-C-A	Protective effect
Lopes et al. (2021)	Case-control	Brazil	Rugby, soccer, combat sports, handball, and water polo		135 Case 135 CON	Tendinopathy	Mixed sports athletes	*TNF-*α	(-1031T>C,-857C>T, −308G>A)	T-C-A	Increased risk
Lulińska-Kuklik et al. (2019b)	Case-control	Polish and Eastern Europe	Soccer and skiing	Males and females	222 ACLR 190 CON	ACL rupture	Polish league players (11–14 h/week)	*VEGFA*	(rs699947- rs1570360-rs2010963)	G-G-C G-C-C	Protective effect Increased risk

ACL: anterior cruciate ligament, RCT: rotator cuff tendinopathy, CTS: carpal tunnel syndrome, CON: controls.

Moderate evidence reflects associations reported in more than one study but showing some inconsistency across populations or study designs. Weak evidence corresponds to preliminary findings derived from a single study, small sample sizes, or insufficient supporting evidence.

Some genes are not presented in Table 1 but are included in Table 2. The corresponding polymorphisms did not show significant associations when analyzed individually; however, they demonstrated significant effects when combined with other polymorphisms, highlighting the importance of gene-gene interactions. This pattern was notably observed for the *LUM*, *IL6R,* and *FGF3* gene.

### 3.1 ECM structure and connective tissue integrity

Genetic variants that affect the structure and integrity of tendon and ligament tissues influence the function of the proteins they encode, potentially increasing susceptibility to these injuries. This includes mutations in collagen genes, as well as disruptions in the expression levels of glycoproteins and proteoglycans, which play crucial roles in the ECM (Larruskain, 2018; Kaynak et al., 2017).

#### 3.1.1 Collagens and structural ECM proteins

Genes encoding different types of collagen play a crucial role in the integrity of tendon and ligament tissues. Fibrillar reorganization, as observed in the genes *COL1A1, COL1A2, COL3A1, COL5A1*, and *COL22A1,* is essential for their proper functioning. Variations in the *COL1A1* gene include −1997A/C (rs1107946), −1663IndelA (rs11327935), and +1245C/A (rs1800012). These variants are located in the promoter region and in intron 1 of *COL1A1*, influencing transcriptional activity by modifying interactions between DNA and transcription factors. Notably, the Sp1-binding site polymorphism (+1245C/A) has been consistently implicated in susceptibility to injury. Carriers of the minor G allele exhibited an approximately 1.43-fold lower risk of ACL rupture compared to T allele carriers in recreational Polish skiers (Stępien-Słodkowska et al., 2013). Furthermore, genotype-specific effects have been reported, with a lower frequency of the GG genotype (rs1800012) observed among ACL rupture cases in male recreational skiers (Stepien-Slodkowska et al., 2017), suggesting a potential role of allelic variation in modulating susceptibility. Based on this protective effect, several studies have investigated the association between this variant and the risk of ligament injury, reporting sometimes conflicting results. For example, the TT genotype is associated with a protective effect against acute soft tissue injuries in cohorts from South Africa and Sweden (Collins et al., 2010), and this association was confirmed by a meta-analysis including 933 cases and 1,381 controls (Wang et al., 2017). Conversely, another study conducted among elite male Australian Football League players showed that the TT genotype was significantly associated with a higher number of total ligament injuries (p = 0.019) and non-contact ligament injuries (p = 0.002) per match (Jacob et al., 2022). Additionally, another study found that exercise addiction may increase injury risk in female volleyball players, while the *COL1A1* rs1800012 T allele appears to be associated with longer recovery durations (Piri et al., 2025). At the haplotype level, a significant underrepresentation of the G-T haplotype (−1997G/+1245T) was observed in individuals with ACL rupture compared with controls (p = 0.048), supporting a combined effect of regulatory variants on injury risk (Ficek et al., 2013). Furthermore, another study conducted in athletes from various sports disciplines identified a protective association against ACL rupture (OR = 0.25; 95% CI = 0.07–0.96) in individuals carrying the rs1107946 (GT or TT) genotypes of *COL1A1,* in combination with wild-type genotypes of three *COL1A2* polymorphisms (rs412777, rs42524, and rs2621215), with the latter two also being independently associated with non-contact ACL injury risk (Perini et al., 2022). The *COL1A2* rs42524 polymorphism is associated with an increased risk of KLR, with the C allele and CC genotype significantly elevating susceptibility. In contrast, for rs2621215, the G allele and GG genotype also act as risk factors, nearly tripling the likelihood of non-contact KLR (Krawczak-Wójcik et al., 2025). However, no independent association between rs1107946 of the *COL1A1* gene and rs1800012 of the *COL1A2* gene and ACL rupture susceptibility was observed in certain cohorts, despite the presence of haplotypic effects (Ficek et al., 2013). Similarly, several independent studies have reported no significant associations between *COL1A1* polymorphisms and tendon or ligament injuries (Larruskain, 2018; Pruna et al., 2013; Shukla et al., 2020; Sivertsen et al., 2019).

Previous studies have reported a significant association between the AA genotype of the *COL3A1* rs1800255 polymorphism and ACL rupture in Polish skiers and professional first-division soccer players (Stępień-Słodkowska et al., 2015a; O’Connell et al., 2015)^.^ However, no significant association between *COL3A1* rs1800255 genotypes and ACL rupture risk was observed in the South African cohort (O’Connell et al., 2015). Similarly, other studies have reported no association between this polymorphism and ACL rupture risk in female elite athletes (Sivertsen et al., 2019; İpekoğlu et al., 2023).

Posthumus was the first to identify a genetic risk factor for anterior ACL rupture in female athletes, linked to the *COL5A1* gene, noting that the CC genotype is more frequent in controls than in individuals with ACL rupture (Posthumus et al., 2009). This finding was further supported by another study, which showed that the CC genotype at *COL5A1* rs12722 is less common in Caucasian females with surgically confirmed ACL ruptures. In contrast, this pattern is not evident in males recruited from sports and recreational settings (Calò et al., 2024). In football, several studies have reported associations between *COL5A1* gene polymorphisms and the risk of ACL rupture. For instance, homozygosity for the C-C rs12722-rs13946 haplotype appears protective against ACL injury (Lulińska-Kuklik et al., 2018). The rs12722 polymorphism is associated with ACL ruptures, with the T allele and TT genotype more frequent among injured players, while the C allele confers approximately a 2-fold reduced risk (İpekoğlu et al., 2023). In a large cohort of South African female players, the CC genotype was associated with a lower risk of ACL rupture (O’Connell et al., 2015), whereas the CC rs13946 genotype was linked to an increased risk in professional female athletes (Rodas et al., 2023). Furthermore, the rs12722 polymorphism has been correlated with the severity of hamstring injuries in elite footballers (Larruskain, 2018). Another study reported that the TT rs12722 genotype is significantly associated with ligament injuries, fewer ankle injuries, and a higher number of matches played (McCabe and Collins, 2018). However, other studies have reported no significant association between rs12722 and ligament injuries (Jacob et al., 2022; Pruna et al., 2013; Krawczak-Wójcik et al., 2025; Stępień-Słodkowska et al., 2015b). Posthumus *et al.* investigated the *COL12A1* gene as a potential risk factor for ACL rupture, including 129 injured individuals and 216 controls. Two single-nucleotide polymorphisms (SNPs), rs240736 and rs970547, were analyzed. The AA genotype of rs240736 was significantly associated with approximately a 2.4-fold increased risk of injury in women, whereas no association was observed for rs970547 (Posthumus et al., 2010). Similarly, a study conducted in a Polish population reported an association between the rs970547 AA genotype and ACL injury risk in females. In addition, the inferred T + A pseudo-haplotype (combining *COL5A1* rs12722 (T/C) and *COL12A1* rs970547 (A/G)) was associated with an increased risk of ACL rupture in female participants from both the South African and Polish cohorts, as well as in the combined analysis (O’Connell et al., 2015). In a study of Polish football players diagnosed with ACL rupture, the frequency of the G allele was lower in injured athletes (18.1%) than in controls (21.3%), although this difference did not reach statistical significance (p = 0.40) (Ficek et al., 2014). However, another study reported no significant association between these polymorphisms and tendon injuries (TEN), ankle injuries (ATI), or rupture (RUP) among South African participants (September et al., 2008). Finally, additional studies conducted in Norwegian, Finnish, Turkish, and broader European cohorts of football, volleyball, and basketball players did not confirm any association between rs970547 and ACL rupture (Sivertsen et al., 2019; Bozkurt et al., 2023). The *COLGALT1* rs8090 polymorphism may contribute to the pathophysiology of connective tissue disorders, potentially by altering post-translational processes that modulate collagen-modifying enzymes. Evidence from a GWAS suggests a possible association between rs8090 and ACL rupture (p = 6 × 10^−4^), although this finding did not reach genome-wide significance and therefore requires further validation (Kim et al., 2017). Additionally, the AA genotype and A allele of rs8090 have been reported to be significantly overrepresented in athletes, particularly rugby union forwards, compared with non-athletes (P ≤ 0.03), indicating a potential link with athlete status (Brazier et al., 2023).

Collagen-related genes, particularly *COL1A1, COL3A1, COL5A1*, and *COL12A1,* show the most recurrent associations with tendon and ligament injuries, especially ACL rupture. However, the results remain inconsistent across studies due to differences in sex, ethnicity, sport type, injury phenotype, and sample size. Associations involving *COL5A1* and *COL12A1* appear more consistent in female athletes, particularly in Caucasian and South African populations, whereas *COL1A1* findings vary between skiers, football players, and volleyball athletes. These discrepancies may reflect population-specific genetic backgrounds and sport-related mechanical demands. In addition, several associations were observed mainly through haplotypes rather than isolated variants, supporting the importance of gene-gene interactions. Overall, the level of evidence is moderate for *COL1A1, COL5A1,* and *COL12A1*, while the evidence for *COLGALT1* remains limited and requires further validation in larger multi-ethnic cohorts.

#### 3.1.2 Proteoglycans and glycoproteins

Proteoglycans and glycoproteins, expressed by numerous genes, are crucial for the proper functioning of the ECM, tissue integrity of tendons and ligaments, as well as the rearrangement of collagen fibrils, such as those encoded by the *ACAN* gene. A study highlighted an association between the rs1516797 GT genotype of the *ACAN* gene and an increased risk of ACL rupture, with a risk ratio of 1.68 in the Polish control group compared with the ACL rupture group. The TT genotype was associated with increased protection (Cięszczyk et al., 2017). However, another study found no significant differences in the genotype frequencies of the *ACAN* polymorphisms rs2351491 and rs1042631 between the control and ACL groups. Interestingly, the TT genotype at rs1516797 was more frequent in the control group, and the T-C-T haplotype was overrepresented in this group, suggesting a potential association with injury susceptibility among sports participants (Mannion et al., 2014). A systematic review on ACL rupture and a study on football players regarding hamstring injury risk found no significant association (Larruskain, 2018; Kaynak et al., 2017). Furthermore, one study showed that the *BGN* rs1042103 A allele was associated with an increased risk of ACL rupture, and that the C-A and T-A *BGN* haplotypes (rs1042103, rs1126499) influenced ACL rupture susceptibility in female and male groups, respectively (Cięszczyk et al., 2017). However, another study found no significant differences in the genotype and allele frequencies of the *BGN* polymorphisms rs1042103 and rs1126499 between the control and ACL groups, although trends suggested a higher frequency of the C-G haplotype (rs1126499-rs1042103) in female controls (Mannion et al., 2014). Additionally, a systematic review reported no significant association (Kaynak et al., 2017)**.** Moreover, a study among football players revealed an association between the A allele of the rs516115 polymorphism in the *DCN* gene and an increased risk of hamstring injuries (Larruskain, 2018). No significant difference was observed in the genotype frequencies of *DCN* SNPs rs13312816 and rs516115 between the control and ACL groups, except for the GG genotype of rs516115, which was more frequent in the control group. Additionally, the AA genotype was underrepresented in the control group (Mannion et al., 2014). A systematic review also found no significant association (Kaynak et al., 2017). The combined analysis of *BGN* (rs1126499-rs1042103), *COL5A1* (rs12722), and *DCN* (rs516115) polymorphisms showed that the C-G-C-G allele combination was associated with reduced ACL injury susceptibility, whereas the T-A/G-T-A combination was linked to increased risk, with sex-specific effects observed, as only the C-A-T-A combination was significantly associated with reduced risk in males (Willard et al., 2018). In a study conducted among Polish football players, the *FMOD* rs7543148 TT genotype was found to be significantly less frequent in the injured group compared to controls, with a similar underrepresentation observed specifically in male athletes, suggesting a potential protective effect against injury (Lulińska et al., 2023). Another highlighted the role of *FMOD* in ECM organization in athletes, suggesting that the increased expression of this gene may explain the greater fragility of the ACL in women compared to men (Johnson et al., 2015). No significant associations were observed between *FMOD* polymorphisms (rs7543148 and rs10800912) and ACL rupture, as neither genotype nor allele frequencies differed between injured and control groups, even after stratification by sex or injury mechanism; however, a specific haplotype involving *LUM* (rs2268578 - rs13312816) and *DCN* (rs516115) genes (T-A-G) was overrepresented in controls, suggesting a potential protective effect (Mannion et al., 2014)*.* Additionally, Makone *et al.* demonstrated that the polymorphism of guanine-thymine dinucleotide repeats in the *TNC* gene is associated with Achilles tendon injuries (ATI), with an overrepresentation of alleles containing 12 and 14 repeats in individuals with injuries, while those with 13 and 17 repeats were underrepresented (Mokone et al., 2005). Furthermore, the A allele of the *TNC* rs2104772 polymorphism was associated with a 1.65-fold higher risk of hamstring injury (Larruskain, 2018). However, no association was shown between the rs2104772 polymorphism and injury severity in football players (Pruna et al., 2013; Pruna et al., 2015). Regarding *FBN2*, the GG genotype was significantly more prevalent in the tendon injury group compared with controls (p = 0.035), and the G allele frequency was significantly higher in both the tendon injury and ACL groups relative to controls (p = 0.017 and p = 0.047, respectively) (Khoury et al., 2015).

Proteoglycan and glycoprotein genes show weak and inconsistent associations with tendon and ligament injuries. Although *ACAN, BGN, DCN, FMOD, TNC*, and *FBN2* have been linked to injury risk in specific cohorts, findings are rarely replicated across studies. Most reported associations are study-specific and often vary by sex, sport discipline, and population, with several findings not replicated in independent cohorts or systematic reviews. *ACAN* and *BGN* variants show mixed effects, including both risk and protective haplotypes, particularly in ACL rupture studies, whereas *DCN* and *FMOD* results are largely inconsistent, with only occasional protective or sex-specific signals. *TNC* and *FBN2* appear more consistently associated with tendon-related phenotypes, such as Achilles and hamstring injuries, but these associations remain based on limited datasets. Overall, the level of evidence for these genes is low to moderate, with strong influence of population heterogeneity and sex-specific effects, highlighting the need for larger, standardized studies to clarify their true contribution to injury risk.

#### 3.1.3 Elastin and ECM structural regulators

Genes encode proteins that play a crucial role in the elasticity of tendons and ligaments, which are essential for flexibility and resistance to injury, including the *ELN* gene. Several SNPs in this gene, located in intronic regions, may be targeted by microRNAs such as miRNA-29, thereby affecting protein expression. Some studies have shown a significant association between the AA genotype (rs2289360) and the incidence and severity of ligament injuries in elite professional football players, particularly a link between the AA genotype and severe injuries (grade 3) and prolonged recovery time (>80 days) (Pruna et al.; Artells et al., 2016; Lulińska et al., 2023; Pruna et al., 2015). Similarly, Jacob *et al.* reported that the G genotype of the rs2289360 polymorphism is associated with ligament and TEN in elite male Australian football players (Jacob et al., 2022). Additionally, Hall *et al.* suggested that the C allele (rs2289360) of the *EMILIN* gene may increase collagenous tissue stiffness, making tendons stiffer in CC homozygotes and thereby elevating the risk of injury by increasing strain during eccentric activities. Conversely, the T allele may protect against certain injuries by enhancing tendon compliance (Hall et al., 2022). One study found no link between rs2289360 and hamstring injuries in athletes (Larruskain, 2018).

*ELN*-related variants show limited, non-replicated associations with injury severity, recovery time, and risk, mainly in football populations. However, findings are inconsistent across studies and largely absent in other cohorts. The overall evidence is weak, with a low level of confidence.

### 3.2 ECM remodeling enzymes and inhibitory regulation

#### 3.2.1 Matrix metalloproteinases (MMPs)

MMPs play a central role in ECM degradation and remodeling, processes that are essential for tissue repair and adaptation. Among these, the *MMP3* gene has been extensively investigated in relation to tendon and ligament injuries, particularly AT. In a South African Caucasian cohort, Raleigh *et al.* reported that the rs679620 GG, rs591058 CC, and rs650108 AA genotypes were associated with an increased risk of AT, highlighting the contribution of *MMP3* genetic variability to tendon pathology. Moreover, the 6 A allele of rs3025058 was suggested to increase AT susceptibility through linkage with the rs679620 G allele. Haplotype analyses further emphasized the complexity of these associations: the A-T-G haplotype (rs679620, rs591058, rs650108) was linked to a reduced risk of AT, whereas interaction between the rs679620 G allele and the *COL5A1* rs12722 T allele significantly increased injury risk (Raleigh et al., 2009). similarly, the T-G-A haplotype (rs591058, rs650108, rs679620) has been associated with an elevated risk of ACL rupture in athletes (Simunic-Briski et al., 2024) However, findings remain inconsistent across populations. In Croatian athletes, the rs650108 GG and rs679620 AA genotypes were associated with increased susceptibility to tendinopathies, while the rs591058 TT genotype showed a non-significant trend toward higher risk after correction for multiple testing. In addition, haplotype analysis indicated that the T-G-A combination may contribute to susceptibility to tendinopathy, whereas the C-A-G haplotype appeared protective (Briški et al., 2021). Expanding beyond single-gene effects, a multi-gene haplotype involving *MMP10* (rs486055), *MMP1* (rs1799750), *MMP3* (rs679620), and *MMP12* (rs2276109) (C-2G-G-G) was associated with a reduced risk of ACL rupture, suggesting a combined influence of *MMP* variants on ligament integrity (Posthumus et al., 2012). In football players, specific polymorphisms within these genes appear to exert distinct influences on injury profiles: the *MMP1* rs1799750 II genotype has been linked to a higher likelihood of injury recurrence, whereas the heterozygous ID genotype may confer a protective effect; the *MMP12* rs2276109 G allele has been associated with an increased risk of recurrent injuries; and the *MMP10* rs486055 T allele, particularly in the CT genotype, has been related to a greater susceptibility to partial ACL ruptures (Łosińska et al., 2025). Furthermore, the TT genotype has been observed more frequently among individuals with ligament rupture or sprain than among non-injured athletes, supporting its potential role in injury susceptibility (Brazier et al., 2025).

Overall, ECM remodeling genes, particularly *MMPs* and their inhibitors (e.g., *MMP3, MMP1, MMP10,* and *MMP12*), show biologically plausible yet highly heterogeneous associations with susceptibility to tendon and ligament injury. Although several *MMP3* variants and haplotypes have been repeatedly linked to Achilles tendon and ACL-related outcomes, findings are inconsistent across populations, with some studies reporting opposite effects or no association. Similarly, other *MMP* family members show context-dependent associations that vary by sport type, injury recurrence, and cohort characteristics. Evidence for these genes is further complicated by strong haplotypic and gene-gene interaction effects, suggesting that isolated SNP analyses may underestimate their contribution. Overall, the level of evidence is moderate at best for *MMP3*, but low and inconsistent for other *MMP* variants, with substantial influence of population heterogeneity, injury definition, and limited replication across independent cohorts.

#### 3.2.2 Tissue inhibitors of metalloproteinases (TIMPs)

TIMPs are key regulators of ECM homeostasis, acting primarily through the inhibition of MMPs. By maintaining the balance between ECM degradation and synthesis, TIMPs contribute to tendon and ligament integrity and play an essential role during tissue repair and remodeling following injury. Among the *TIMP* family members, *TIMP2* has been investigated in relation to musculoskeletal injury susceptibility. The *TIMP2* rs4789932 polymorphism has been associated with Achilles tendon pathology in Caucasian populations, with the TT genotype has been associated with an increased risk of Achilles tendon pathology in Caucasian populations (El Khoury et al., 2013). In contrast, no significant association between the *TIMP2* rs4789932 polymorphism and ACL rupture susceptibility was observed among Polish soccer players (Lulińska-Kuklik et al., 2019c).

Overall, the available evidence suggests a possible role of *TIMP2* in tendon injury susceptibility, however, findings remain limited and inconsistent across different injury phenotypes. Therefore, the current level of evidence is low, reflecting the small number of studies and the lack of independent replication.

#### 3.2.3 ADAMTs family

The *ADAMTS14* gene, which plays a role in ECM remodeling and collagen organization, has been investigated in relation to musculoskeletal injury susceptibility. However, evidence remains limited, with only a small number of studies evaluating its association with injury risk. Among these, one study in football players reported that the AG genotype of the *ADAMTS14* rs4747096 polymorphism was associated with an increased risk of both acute and overuse hamstring injuries (Larruskain, 2018). In contrast, another study found no association between this polymorphism and Achilles tendon pathology in Caucasian participants (El Khoury et al., 2013).

Overall, current findings regarding *ADAMTS14* remain inconsistent and limited, highlighting the need for further well-powered studies to clarify its potential role in injury susceptibility.

### 3.3 Inflammation and immune response in tissue injury

The immune response, which plays a pivotal role in tissue repair following injury, is initiated by an inflammatory reaction mediated by signaling molecules such as chemokines and cytokines. These molecules activate and recruit immune cells to the site of injury and are regulated by genes including *IL1B*, *IL6*, and *IL6R*. Among the polymorphisms investigated, *IL6* rs1800795, *IL6R* rs2228145, and *IL1B* rs1143634, evidence from studies in football players indicates that the *IL6* rs1800795 GG genotype is consistently associated with an increased risk of both hamstring injuries and ACL rupture (OR = 1.68 and 1.74, respectively), whereas the heterozygous GC genotype appears to exert a protective effect against ACL injury (Larruskain, 2018; Lulińska-Kuklik et al., 2019a). In contrast, a study including athletes from non-contact jumping sports, non-contact non-jumping sports, and skiing reported no significant association between *IL6* rs1800795 or *IL6R* rs2228145 polymorphisms and ACL injury risk. However, the *IL1B* rs16944 TT genotype has been associated with approximately a 3-fold increased risk of non-contact ACL rupture in female participants. Furthermore, gene-gene interaction analysis involving *COL5A1* rs12722 (T/C), *IL1B* rs16944 (C/T), *IL6* rs1800795 (G/C), and *IL6R* rs2228145 (A/C) demonstrated that the T-C-G-A allele combination was significantly underrepresented in controls and more frequent in both ACL rupture and non-contact subgroups, suggesting increased injury susceptibility (Rahim et al., 2017). Additionally, a recent meta-analysis identified *IL6* rs1800795 as significantly associated with ACL rupture, with the GC genotype linked to increased risk, while the CC genotype of *IL6* rs1800795 and the CT genotype of *IL1B* rs16944 were associated with a protective effect (Lorenz et al., 2024). Polymorphisms in the *TNF-*α gene, particularly the variant allele −308 A and T-C-A haplotype (−1031T>C, −857C>T, and −308G>A), have also been associated with an increased risk of tendinopathies in athletes (Lopes et al., 2021). However, no association was observed between *TNF-*α rs1800629 (−308 G/A) and hamstring injuries in football players or ACL rupture in athletes from non-contact jumping sports, non-contact non-jumping sports, and skiing (Larruskain, 2018; Rahim et al., 2017). Furthermore, the presence of the *FCRL3* -169C allele was associated with an increased risk of tendinopathy, while the combined variant genotypes *FCRL3* -169TC or −169CC and *FOXP3* -2383CT or −2383TT were also linked to an increased risk of tendinopathy in athletes with tendon pain, particularly among volleyball players (Salles et al., 2018). At the molecular level, the *CASP8* rs3834129 (−652 6N del) polymorphism disrupts a Sp1 transcription factor binding site, leading to reduced *CASP8* transcription, while rs1045485 (Asp302His) may affect caspase-8 protein-protein interactions. *CASP8*, a key regulator of apoptosis, is also modulated by *IL6* signaling and plays an important role in programmed cell death pathways (Rahim et al., 2017; Seale et al., 2020). Regarding genetic associations, Rahim et al. reported that the *CASP8* rs3834129 insertion allele and the rs1045485 GC genotype were associated with an increased risk of ACL rupture. Moreover, the *CASP8* del-G haplotype (rs3834129 (ins/del)-rs1045485) was significantly underrepresented in controls compared with both ACL rupture and non-contact injury groups, suggesting a risk-increasing effect (Rahim et al., 2017). Consistently, haplotype analysis combining the *CASP8* polymorphisms rs3834129 (ins/del), rs1045485 (G/C), and rs13113 showed significant associations with ACL injury susceptibility, despite no individual SNP effects, with the ins-G-T haplotype (rs3834129-rs1045485-rs13113), as well as the ins-T (rs3834129-rs13113) and G-T (rs1045485-rs13113) combinations, associated with increased risk, whereas the ins-G-A (rs3834129-rs1045485) and del-C (rs3834129-rs1045485) haplotypes were associated with a protective effect against ACL rupture and non-contact ACL injuries (Seale et al., 2020). In contrast, the rs3834129 polymorphism in the *CASP8* gene was not associated with the risk of hamstring injuries in football players (Larruskain, 2018).

Overall, the variants (notably *IL6* rs1800795 and *IL1B* rs16944) appear repeatedly linked to ACL rupture and hamstring injuries, particularly in football players and in female-specific analyses. Other studies report null or inconsistent results across mixed-sport and multi-ethnic cohorts. Evidence for *TNF-*α and *CASP8* is mainly derived from limited studies and haplotype-based analyses, suggesting that gene-gene interactions may be more informative than single polymorphisms. Overall, the level of evidence ranges from moderate for *IL6/IL1B* to low for the other inflammatory genes, with strong influence of sex, sport type, and population heterogeneity, and limited replication across independent cohorts.

### 3.4 Growth factors and signaling pathways involved in tissue repair and regeneration

Several genes encode growth factors involved in tissue repair, such as the *GDF5*. Players with the TT genotype (rs143383) of the *GDF5* gene are at an increased risk of ankle and knee injuries, as well as a reduction in the number of matches played. This trend is also observed among semi-professional and amateur players, but not among professionals, suggesting that normal levels of *GDF5* may not be sufficient to prevent injuries in these groups (McCabe and Collins, 2018). Furthermore, no association was found between the rs143383 polymorphism in the *GDF5* gene and the risk of hamstring injuries in football players (Larruskain, 2018). Similarly, the GG genotype of *IGF2* (rs680) is associated with increased injury risk and fewer matches played, a trend also observed in semi-professional and amateur players (McCabe and Collins, 2018). In contrast, the GC genotype (rs3213221) is associated with fewer severe injuries than the GG or CC genotypes (Pruna et al.). The *IGF2* rs3213221-CC genotype was strongly associated with a higher estimated number of contact TEN per match, as well as an increased number of total ligament injuries and non-contact ligament injuries (Jacob et al., 2022)^.^ However, no association was found between *IGF2* rs3213221 and hamstring injuries in football players (Larruskain, 2018). Salles *et al.* reported that volleyball athletes carrying the *BMP4* rs2761884 GT + TT genotypes had a 2.4-fold increased susceptibility to tendinopathy during training. In addition, the *BMP4* TTGGA haplotype (rs2761884, rs17563, rs2855529, rs2071047, rs762642) was associated with tendinopathy occurrence, while the *FGF3* TGGTA haplotype (rs7932320, rs1893047, rs12574452, rs4631909, rs4980700) showed a suggestive trend toward association with tendinopathy (Salles et al., 2015).

Overall, growth factor genes involved in tissue repair, including *GDF5, IGF2, BMP4*, and members of the *FGF* family, show suggestive but inconsistent associations with susceptibility to tendon and ligament injury. While certain variants, such as *GDF5* rs143383, *IGF2* rs680/rs3213221, and *BMP4* haplotypes, have been linked to injury risk, severity, or reduced performance in specific cohorts (particularly football and volleyball players), these findings have not been consistently replicated across populations and sports disciplines. Several associations also differ by competitive level, with stronger effects reported in semi-professional and amateur athletes compared to professionals, suggesting a potential role for training adaptation and exposure. In addition, some variants show opposite or null effects depending on the injury phenotype analyzed (e.g., hamstring vs. ligament injuries). Overall, the level of evidence is low to moderate, with substantial heterogeneity driven by sport type and performance level, and limited replication, highlighting the need for larger and more standardized studies to clarify the role of growth factor genes in musculoskeletal injury risk.

### 3.5 Angiogenesis and vascular regulation in tendon and ligament healing

Several genes regulate the vascularization and angiogenesis of tendons and ligaments. These processes play a crucial role in injury recovery by activating signaling pathways that stimulate new blood vessel formation and oxygen transport, both of which are essential for proper tissue function. Genetic variants within these genes can modulate the efficiency of vascular responses and tissue repair, potentially influencing injury susceptibility and recovery outcomes (Salles et al., 2016; Rahim et al., 2014; Rahim et al., 2018).

#### 3.5.1 *VEGFA* and *VEGFR2* signaling

Vascular growth and angiogenesis-related processes are regulated by genes such as *KDR/VEGFR2* and *VEGFA*, which have been investigated for their potential roles in susceptibility to tendon and ligament injury. In volleyball players, the *KDR* 1192G>A polymorphism, particularly the GA and GA + AA genotypes, as well as the CGA and CAT haplotypes (−604C>T, 1192G>A, 1719T>A), have been associated with a reduced risk of tendinopathy (Salles et al., 2016). In contrast, the *KDR* rs2071559 polymorphism has shown inconsistent associations with injury risk: the AG genotype was more frequent in controls, whereas the GG genotype was less frequent in control groups compared with ACL rupture cases, suggesting a possible role in susceptibility (Rahim et al., 2018). However, a study in South African Caucasians reported no significant association between individual *KDR* variants (rs1870377, rs2071559) and ACL rupture, although haplotype analysis revealed that the G-A haplotype was consistently underrepresented in controls compared with both ACL rupture and non-contact injury groups, indicating a potential association with increased injury susceptibility across sexes (Rahim et al., 2014). The *VEGFA* gene has also been widely studied in relation to musculoskeletal injuries. In Indian athletes, the rs699947 A allele and the rs35569394 I allele were associated with an increased risk of ACL injury (M et al., 2020). In Caucasian populations, the rs699947 CC genotype was associated with a higher risk of non-contact ACL rupture, whereas the rs1570360 GA genotype appeared protective. Consistently, haplotype analyses of *VEGFA* variants (rs699947, rs1570360, rs2010963) highlighted differential associations with injury risk: the A-A-G haplotype was more frequent in controls than in the non-contact subgroup. In contrast, the C-G-C haplotype was underrepresented in female controls, suggesting sex-specific genetic effects (Rahim et al., 2014). Similarly, among Polish Caucasians, the rs2010963 C allele was associated with ACL rupture, whereas rs699947 and rs1570360 showed no individual effects; nevertheless, haplotype analysis identified the C-G-G haplotype as protective and the C-G-C haplotype as risk-associated for ACL rupture (Lulińska-Kuklik et al., 2019b). In contrast, no association was observed between *VEGFA* polymorphisms and tendinopathy in volleyball players or Achilles tendinopathy in South African populations (Salles et al., 2016; Rahim et al., 2018). Finally, the *WISP2/CCN5* gene, which is expressed at lower levels in women, has been suggested to contribute to increased ACL injury susceptibility by influencing ligament composition and structural integrity, and may potentially contribute to increased susceptibility to rupture (Johnson et al., 2015).

*VEGFA* shows a relatively stronger and more consistent association with injury susceptibility, although results vary across populations (Indian, Polish, and Caucasian) and sport disciplines. In contrast, *KDR* associations, studied notably in volleyball and other sports, are more heterogeneous and less consistently replicated. Overall, the findings are strongly influenced by population- and sport-specific factors, with haplotype analyses providing more robust signals than single-variant analyses. The overall level of evidence remains moderate, with *VEGFA* showing stronger support than *KDR*.

#### 3.5.2 Hypoxia and oxygen regulation pathways (*HIF1A*)

Genetic variants involved in oxygen homeostasis and angiogenesis, particularly within the *HIF1A* gene, have been investigated for their potential functional relevance in sports injuries. In football players, the rs11549465 CC genotype has been associated with an increased risk of hamstring injuries, with carriers of this genotype showing approximately a 2-fold higher likelihood of injury compared with CT individuals (Larruskain, 2018). In contrast, a study conducted among physically active South African Caucasians reported no significant association between the rs11549465 polymorphism and ACL injury risk (Rahim et al., 2014).

*HIF1A* rs11549465 shows a possible association with hamstring injury in football players, but this has not been replicated in other populations or injury types. Overall, the evidence is limited and remains low.

### 3.6 Muscle function and mechanical load adaptation

Alterations in muscle function, particularly in contraction efficiency, can contribute to the development of TEN, while ligaments may also be compromised by the high mechanical loads generated during muscular activity. Moreover, muscle fatigue has been implicated in both ligament and tendon damage, partly as a result of metabolic byproduct accumulation within the ECM (Varillas-Delgado, 2024; McCabe and Collins, 2018). Genetic factors, particularly the *ACTN3* gene, may influence muscle contraction efficiency and fatigue resistance during high-intensity activity. In Korean ballet dancers, the XX genotype has been associated with reduced flexibility compared with RR and RX genotypes, likely due to the absence of α-actinin-3, which may impair fast-twitch muscle function and connective tissue support. This genotype may therefore affect performance and injury susceptibility, suggesting that individuals with the XX variant could benefit from targeted strength training to improve functional capacity and reduce injury risk (Kim et al., 2014). Runners carrying the RR genotype had a significantly higher proportion of Achilles tendon injuries (p = 0.025). In contrast, no association was observed between *ACTN3* polymorphisms and the overall distribution of tendon or ligament injury types (Gutiérrez-Hellín et al., 2021).

However, several other studies have reported no significant association between the *ACTN3* polymorphism and sports injury risk (Del Coso et al., 2022; Onori et al., 2022; Var et al., 2023). Furthermore, in football players, both studies consistently suggest a protective effect of the *AMPD1* CC genotype, which is associated with a lower incidence of ankle and knee injuries and greater match participation, while the TT genotype appears more frequent among injured athletes and was associated with a higher occurrence of injuries, suggesting a potential association with increased injury susceptibility (Varillas-Delgado, 2024; McCabe and Collins, 2018). Additionally, carriers of the T allele, who often experience prolonged recovery periods after exercise, may be more susceptible to injuries due to inadequate rest (Collins, 2017).

*ACTN3* and *AMPD1* genes show variable associations with injury risk in runners and with flexibility in Korean ballet dancers. *ACTN3* findings are inconsistent and lack strong support across studies, whereas *AMPD1* shows more reproducible associations in football players. Overall, the level of evidence remains low to moderate and is influenced by sport type and population differences.

### 3.7 GWAS findings in tendon and ligament injuries

GWAS investigating lower-limb musculoskeletal injuries remain scarce and largely exploratory, yet they provide important insights into the highly polygenic architecture of Achilles tendon pathology, ACL rupture, and tendinopathies (Ebert et al., 2023; Kim et al., 2017). In elite athletic cohorts, including Olympic competitors and members of the Estonian national team, one study of 47 injured individuals reported 37 SNPs reaching genome-wide significance for injury susceptibility across 32 genes (Ebert et al., 2023). However, the extremely limited sample size and discovery-driven design substantially increase the risk of false-positive associations, necessitating cautious interpretation. A second GWAS in patients with Achilles tendon pathology, ACL rupture, and tendinopathies identified two additional genome-wide significant loci. Still, none have been replicated in athletic populations, underscoring their preliminary nature. Collectively, the GWAS signals summarized in Table 3 should therefore be considered hypothesis-generating findings with weak evidential support, pending replication in independent and sport-specific cohorts (Kim et al., 2017). In contrast to candidate-gene studies, which are hypothesis-driven and anchored in established biological pathways such as collagen turnover and ECM remodeling, GWAS use an agnostic, genome-wide scanning strategy that is inherently susceptible to spurious associations in underpowered datasets. Accordingly, robust interpretation of GWAS findings requires independent replication and functional validation before biological or clinical relevance can be inferred.

**TABLE 3:** Genes implicated in ligament and tendon injuries.

Genes	Full name	Polymorphisms	Localization
*CDHR2* (Ebert et al., 2023)	Cadherin-related family member 2	rs76277542	5q35.2
*CIT* (Ebert et al., 2023)	Citron rho-interacting serine/threonine kinase	rs77411677	12q24.23
*COL22A1* (Ebert et al., 2023)	Collagen type XXII alpha 1 chain	rs3924862 rs79329230	8q24.23-q24.3
*CRX* (Ebert et al., 2023)	Cone-rod homeobox	rs8102250	19q13.33
*CYTH4* (Ebert et al., 2023)	Cytohesin 4	rs1041895	22q13.1
*DAP* (Ebert et al., 2023)	Death-associated protein	rs267959 rs2930047 rs1080440 rs267939	5p15.2
*DOK5* (Ebert et al., 2023)	Docking protein 5	rs73142922	20q13.2
*DST* (Ebert et al., 2023)	Dystonin	rs73749948	6p12.1
*EPCAM-DT* (Ebert et al., 2023)	EPCAM divergent transcript	rs13401365	2p21
*GNG12* (Ebert et al., 2023)	G Protein subunit gamma 12	rs28435277	1p31.3
*GRIK4* (Ebert et al., 2023)	Glutamate ionotropic receptor kainate type subunit 4	rs3133223	11q23.3
*HEPACAM2* (Ebert et al., 2023)	HEPACAM family member 2	rs17165143	7q21.2
*ITIH2* (Ebert et al., 2023)	Inter-alpha-trypsin inhibitor heavy chain 2	rs7913729	10p14
*KIAA1217* (Ebert et al., 2023)	KIAA1217	rs2297329	10p12.2-p12.1
*LDLRAD2* (Ebert et al., 2023)	Low-density lipoprotein receptor class A domain containing 2	rs67141521	1p36.12
*LINGO2* (Ebert et al., 2023)	Leucine-rich repeat and Ig domain containing 2	rs4305988	9p21.2-p21.1
*LOC101927609/LOC107986772* (Ebert et al., 2023)	Uncharacterized LOC101927609 Uncharacterized LOC107986772	rs10950647	7p21.1
*LOC105377509* (Ebert et al., 2023)	Uncharacterized LOC105377509	rs1481217	4q32.1
*LOC107985411* (Ebert et al., 2023)	Uncharacterized LOC107985411	rs6053422	20p12.3
*LOC107987166* (Ebert et al., 2023)	Uncharacterized LOC107987166	rs488435	11q23.3
*LOC730100/LOC124907767* (Ebert et al., 2023)	Uncharacterized LOC124907767	rs79526092	2p16.3
*MAS1* (Ebert et al., 2023)	Proto-oncogene, G protein-coupled receptor	rs220735 rs170219	6q25.3
*MIR608* (Kim et al., 2017)	MicroRNA 608	rs4919510	10q24.31
*NID1* (Kim et al., 2017)	Nidogen 1	rs4660148	1q42.3
*OSBPL10* (Ebert et al., 2023)	Oxysterol binding protein like 10	rs6800757	3p23-p22.3
*PAPPA2* (Ebert et al., 2023)	Pappalysin 2	rs11580456	1q25.2
*PIEZO2* (Ebert et al., 2023)	Piezo type mechanosensitive ion channel component 2	rs140107052	18p11.22-p11.21
*PLXNA2* (Ebert et al., 2023)	Plexin A2	rs11799530	1q32.2
*SLC25A43* (Ebert et al., 2023)	Solute carrier family 25 member 43	rs11798653	Xq24
*SMCO2* (Ebert et al., 2023)	Single-pass membrane protein with coiled-coil domains 2	rs385878	12p11.23
*ST6GAL1* (Ebert et al., 2023)	ST6 beta-galactoside alpha-2,6-sialyltransferase 1	rs4012250	3q27.3
*TSPAN2* (Ebert et al., 2023)	Tetraspanin 2	rs41274142	1p13.2
*VRTN* (Ebert et al., 2023)	Vertebrae development associated	rs2286425	14q24.3
*ZNF800* (Ebert et al., 2023)	Zinc finger protein 800	rs111476429	7q31.33

## 4 Modifiers of genetic susceptibility

### 4.1 Population heterogeneity and sex-specific genetic effects

An important consideration in interpreting genetic associations with tendon and ligament injuries is the substantial heterogeneity across populations, sexes, sports disciplines, competitive levels, and injury phenotype. Many of the reported associations appear to be population-specific and are not consistently replicated across independent cohorts. Several polymorphisms, particularly within *COL5A1, COL12A1, VEGFA, KDR,* and *MMP3,* demonstrate sex-specific or ethnicity-dependent effects. For example, associations involving *COL5A1* rs12722 and *COL12A1* rs240736/rs970547 were more consistently observed in female athletes and in South African or Caucasian populations. In contrast, findings in mixed-sex or other ethnic cohorts were often inconsistent or absent. Similarly, *VEGFA* haplotypes showed sex- and population-background-specific associations, while *MMP3* associations varied across the Achilles tendinopathy, ACL rupture, and tendinopathy cohorts. These discrepancies may reflect differences in genetic background, linkage disequilibrium structure, hormonal influences, biomechanical loading patterns, training exposure, injury mechanisms, and sport-specific demands. In addition, several studies included relatively small cohorts, increasing the risk of population stratification bias and false-positive findings. Therefore, current genetic associations should not be generalized across all athletic populations or injury types. Future studies should prioritize larger multi-ethnic cohorts, standardized injury definitions, sex-stratified analyses, and replication across independent populations to improve the robustness and generalizability of findings.

### 4.2 Multifactorial interaction between genetics and extrinsic/intrinsic factors in tendon and ligament injuries

Tendon and ligament injuries result from a multifactorial model in which genetic predisposition interacts dynamically with both intrinsic and extrinsic factors. Genetic variants involved in ECM organization, particularly those in collagen genes (e.g., *COL1A1, COL5A1*) and proteoglycan genes (e.g., *ACAN, DCN*), influence the mechanical properties of connective tissues, including tensile strength, elasticity, and load tolerance. These genetic effects become particularly relevant in the context of extrinsic factors such as training volume, intensity, and periodization, where excessive or poorly managed loads may significantly increase injury risk in individuals carrying susceptibility-associated variants (Jacob et al., 2022; Leźnicka et al., 2021; Krawczak-Wójcik et al., 2025; İpekoğlu et al., 2023).

In addition, a history of injury is a major risk factor for recurrence. It may interact with polymorphisms involved in tissue repair and ECM remodeling, thereby influencing both the duration and quality of recovery. Intrinsic factors such as sex, age, and biological maturation further modulate injury susceptibility through hormonal, structural, and developmental differences in musculoskeletal tissues, which may amplify or attenuate the biological effects of genetic variation (El Khoury et al., 2013; Raleigh et al., 2009; Briški et al., 2021; Simunic-Briski et al., 2024; Lulińska-Kuklik et al., 2019c).

Moreover, biomechanical factors, including movement technique, repetitive joint loading, playing surface characteristics, and sports equipment, can alter the mechanical stress distribution across tissues and interact with genetic profiles related to muscle function and fatigue, such as *ACTN3* and *AMPD1*, thereby influencing neuromuscular efficiency and injury risk (Marrouh et al., 2025). Circadian rhythm-related genes, such as *PER3*, may also affect performance, recovery, and neuromuscular coordination by interacting with sleep patterns and training schedules, thereby contributing to inter-individual variability in injury susceptibility (von Rosen et al., 2017; Kunorozva et al., 2017; Anderson et al., 2018).

Finally, lifestyle-related factors, particularly sleep quality and nutrition, play a critical role in tissue regeneration and may modulate gene expression through epigenetic and nutrigenomic mechanisms (Mason et al., 2023). In this context, tendon and ligament injuries should be considered the outcome of a complex interplay among genetic, biomechanical, and environmental determinants, underscoring the need for integrated prevention and rehabilitation strategies that account for training load, injury history, biological characteristics, and recovery conditions to optimize return-to-sport outcomes in athletes.

## 5 Importance and limitations of gene-gene interaction and haplotype analyses in tendon and ligament injury susceptibility

Gene-gene interactions and haplotype-based approaches are key components in understanding the genetic susceptibility to tendon and ligament injuries, as they extend beyond the limited explanatory power of individual variants. Indeed, SNPs generally exhibit modest effect sizes and are insufficient to capture the full complexity of the polygenic architecture underlying these conditions. In contrast, haplotypes provide a more integrative framework by capturing combinations of linked variants within a single genomic region, reflecting linkage disequilibrium patterns that may better represent the underlying biological effects on key pathways such as ECM remodeling, inflammation, and tissue repair. Similarly, gene-gene interaction analyses may help explain inter-individual variability in injury susceptibility by accounting for the combined effects of multiple loci that share biological processes. However, despite their biological relevance, these approaches present several important methodological limitations. Haplotype and interaction analyses typically require large sample sizes to achieve sufficient statistical power and are highly sensitive to multiple testing, increasing the risk of false-positive findings if appropriate statistical corrections are not applied. Moreover, many reported associations are population- or sport-specific and are not consistently replicated in independent cohorts, limiting their robustness and generalizability. Finally, their clinical translation remains limited due to the complexity of integrating multi-locus models into standardized predictive frameworks. Therefore, although haplotype and gene-gene interaction analyses provide valuable insight into the polygenic nature of tendon and ligament injuries, they should be interpreted as complementary to single-variant analyses and considered exploratory until validated in large, independent, well-powered studies.

## 6 Limitations

Despite providing a comprehensive synthesis of the current literature on genetic susceptibility to tendon and ligament injuries in athletes, this review has several limitations that should be acknowledged. First, as a narrative review, it does not rely on a fully systematic methodology; therefore, the study selection and interpretation processes may be subject to selection bias and may not reflect all available evidence in the literature. Although a structured search strategy was applied across multiple databases, the absence of a formal systematic methodology limits the reproducibility of the review process. In addition, substantial heterogeneity exists among the included studies regarding injury definitions, sport disciplines, competitive levels, sex distribution, ethnicity, training exposure, and methodological approaches, making direct comparisons difficult. Many studies also relied on relatively small sample sizes and lacked replication in independent cohorts, thereby increasing the risk of inconsistent associations or false-positive findings. Furthermore, most available studies used observational case-control designs, which do not allow causal inference. The potential risk of publication bias should also be considered, as positive findings are more likely to be published. Finally, although several polymorphisms have been associated with injury susceptibility, the predictive value and clinical relevance of individual variants remain limited due to the multifactorial and polygenic nature of tendon and ligament injuries.

## 7 Clinical and practical implications

From a clinical and applied perspective, current genetic evidence in tendon and ligament injuries should be interpreted with caution and not used in isolation for risk stratification. Although genetic research provides valuable insights into the biological mechanisms underlying tissue susceptibility, the available findings are not sufficiently robust or consistent to support the implementation of genetic testing as a standalone predictive tool in sports practice. Instead, genetic information may contribute as one component of broader integrative models aimed at understanding injury risk profiles in athletes. Such models should combine genetic background with established determinants of injury, including biomechanical load, training exposure, previous injury, recovery capacity, and individual psychosocial and physiological characteristics. In addition, the use of genetic information in sports raises important ethical considerations. These include issues related to the privacy and confidentiality of genetic data, the risk of misinterpreting probabilistic associations as deterministic predictions, and the potential for discrimination or inappropriate use in athlete profiling. Therefore, any future application of genetic markers in sports medicine must adhere to strict ethical and regulatory frameworks, ensuring that genetic data are used solely for health promotion, injury prevention, and evidence-based individualized care, rather than for the selection or exclusion of athletes. In this context, the main added value of genetic data lies in improving mechanistic understanding and potentially refining multidimensional risk assessment frameworks, rather than providing direct clinical prediction. Therefore, future applications in sports medicine should focus on multifactorial, data-driven approaches that integrate genetic and non-genetic factors to support injury prevention and individualized athlete management better.

## 8 Conclusion

In conclusion, this review highlights the growing importance of genetic factors in understanding tendon and ligament injuries in athletes. A total of 70 genes and 107 variants have been reported in the literature, including 33 candidate genes with 43 polymorphisms; 25 polymorphisms evaluated in haplotype analyses involving these candidate genes as well as three additional genes, and 34 genes identified through GWAS, encompassing 39 variants, reflecting a complex and multifactorial genetic architecture. Some markers appeared protective in the studied cohort, such as the TT genotype (rs1516797) of *ACAN*, the CC genotype (rs12722) of *COL5A1*, the T allele (rs2289360) of *EMILIN*, the CT genotype (rs16944) of *IL1B*, the TT genotype (rs7543148) of *FMOD*, and the CC genotype (rs17602729) of *AMPD1*. Conversely, other polymorphisms appeared to be associated with tendon and ligament injuries in athletes, such as the ins-G-T haplotype of *CASP8* (rs3834129-rs1045485-rs13113), the TT genotype (rs143383) of *GDF5*, the A-T-G haplotype of the *MMP3* gene (rs676920, rs591058, rs650108), the C-G-C and G-C-C haplotype (rs699947, rs1570360, rs2010963) of *VEGFA*, and the CC genotype rs11549465 of *HIF1A*. However, some polymorphisms yield conflicting results across studies, highlighting the need for further research to reach more robust conclusions. This is particularly applicable for the three polymorphisms (rs676920, rs650108, and rs591058) of the *MMP3*, (rs1800012) polymorphism of *COL1A1*, and (rs699947) of *VEGFA*. Genetic research on tendon and ligament injury prevention remains underexplored, with some associations requiring further validation, particularly in adapting to mechanical stress. Tendon and ligament injuries, including tendinopathy, tendon rupture, ligament injury, ACL rupture, hamstring injury, and ankle injury, represent distinct clinical and biological entities. Therefore, findings should be interpreted within the context of the specific injury phenotype investigated and should not be generalized across all musculoskeletal injuries. Future studies should emphasize large-scale, multi-population research and gene-environment interactions. Integrating multi-omics approaches could enable personalized prevention strategies, optimizing training and recovery, and reducing injury risk in athletes.
